# Efficacy and Safety of the Third‐Generation Tyrosine Kinase Inhibitor Olverembatinib in Combination With Inotuzumab Ozogamicin for the Treatment of Adult Philadelphia Chromosome‐Positive Acute Lymphoblastic Leukemia Patients With Refractory/Relapsed Disease or Persistent Minimal Residual Disease Bridging to Hematopoietic Stem Cell Transplantation

**DOI:** 10.1002/ajh.70026

**Published:** 2025-08-06

**Authors:** Xiaoyu Zhang, Yigeng Cao, Jialin Wei, Weihua Zhai, Qiaoling Ma, Chen Liang, Xin Chen, Wenbin Cao, Donglin Yang, Aiming Pang, Yi He, Sizhou Feng, Mingzhe Han, Rongli Zhang, Erlie Jiang

**Affiliations:** ^1^ State Key Laboratory of Experimental Hematology, National Clinical Research Center for Blood Diseases, Haihe Laboratory of Cell Ecosystem, Institute of Hematology & Blood Diseases Hospital Chinese Academy of Medical Sciences & Peking Union Medical College Tianjin China; ^2^ Tianjin Institutes of Health Science Tianjin China

**Keywords:** allo‐HSCT, immunotherapy, olverembatinib, Ph + ALL

To the Editor,

1

Despite significant advancements in the treatment of Philadelphia chromosome‐positive acute lymphoblastic leukemia (Ph + ALL), particularly the development of tyrosine kinase inhibitors (TKIs), the prognosis of refractory/relapsed (R/R) or persistent minimal residual disease (MRD)‐positive Ph + ALL remains poor. For these patients, allogeneic hematopoietic stem cell transplantation (allo‐HSCT) remains the main approach for long‐term disease‐free survival [1]. However, post‐transplantation relapses remain a major challenge. The remission depth is strongly associated with HSCT outcomes, highlighting the importance of achieving complete molecular remission (CMR) prior to transplantation [2]. The combination of TKIs and immune therapies has been the mainstay of therapy for Ph + ALL. The advent of targeted therapies such as the CD22 monoclonal antibody inotuzumab ozogamicin (INO) and the CD19 bispecific T‐cell Engager (Blinatumomab, BiTE) has renewed interest in strategies to enhance disease remission while minimizing the cytotoxic effects of conventional chemotherapy [3, 4]. These advancements offer promising avenues for improving outcomes in high‐risk patients.

Olverembatinib, currently the only third‐generation TKI available in mainland China, has demonstrated significant efficacy in treating chronic myeloid leukemia (CML) patients with the T315I mutation who are resistant to other TKIs [5]. However, its role in the treatment of R/R or MRD‐positive Ph + ALL, particularly in heavily pretreated patients bridging to transplantation, remains underexplored. To address this gap, we conducted a prospective study to evaluate the efficacy and safety of combining olverembatinib with INO as a bridging therapy prior to transplantation. This approach aims to achieve deeper molecular remission, improve transplantation outcomes, while minimizing treatment‐related toxicity in this high‐risk patient population.

We conducted two open‐label, single‐center, investigator‐initiated phase II studies in Ph/BCR‐ABL1+ ALL patients using this combined treatment but with distinct patient populations. The first study (NCT05603156) enrolled patients with persistent MRD after at least three rounds of chemotherapy, while the second study (ChiCTR2200061432) focused on refractory/relapsed patients. Apart from requirements of primary disease diagnosis and evaluation, patients were required to be older than 16 years and have ≥ 20% of blasts positive for CD22 expression. In both studies, eligible patients received therapy including olverembatinib (40 mg QOD, d1‐28) combined with INO (0.6 mg/m^2^, d1, d8 per 28‐day cycle). Bone marrow MRD was assessed at the end of each treatment cycle. Enrolled patients received a maximum of two treatment cycles before proceeding to HSCT. Post‐transplant maintenance treatment with olverembatinib was administered after adequate hematopoietic recovery was achieved under physician supervision.

The clinical outcomes from both studies were subsequently pooled for comprehensive analysis. The two studies were approved by the Ethics Committee of the Institute of Hematology and Blood Diseases Hospital and were conducted in accordance with the guidelines of the Declaration of Helsinki. All patients or their guardians provided written informed consent prior to enrollment. Response assessments were performed according to the National Comprehensive Cancer Network guidelines for acute lymphoblastic leukemia (version 2, 2021). Recurrence‐free survival was defined as the time from complete remission after transplantation until relapse, death, or the last follow‐up date. Overall survival (OS) was defined as the time from the start of treatment to death or the last follow‐up. Adverse events were graded according to the National Cancer Institute Common Terminology Criteria for Adverse Events, version 5.0. BCR‐ABL kinase domain mutation analysis was performed via direct Sanger sequencing. Patient characteristics were compared via the chi‐square test or Fisher's exact test for binary variables and the Mann–Whitney U test for continuous variables. Survival probabilities were assessed via the Kaplan–Meier method. Statistical analysis was conducted with SPSS version 24.0 software (SPSS Inc., Chicago, IL, USA) and R software (version 2.14.1; http://www.r‐project.org).

The baseline characteristics, treatment details, and responses of the 14 enrolled patients are detailed in Table 1 and Figure 1. Among these patients, five had hematological relapses, eight were persistently positive for MRD, and one had relapsed MRD. Three patients did not respond to at least two different TKIs. All three patients with hematological recurrence achieved a CMR only after one cycle of treatment. Ten of the 14 patients treated for MRD clearance achieved a CMR, resulting in an overall CMR rate of 76.47%. All three patients for whom treatment for MRD clearance failed received only one course of treatment. Only one patient received two cycles of treatment and achieved MRD negativity.

**TABLE 1:** Baseline characteristics of patients.

Characteristic	*N* = 14 (%)
Gender male/female	8 (57.1)/6 (42.9)
Age, median (range), year	42 (21, 65)
WBC at diagnosis, median (range) (×10^9^ cells per L)	7.6 (2.8, 426)
Type of BCR/ABL	
*P190/P210*	8 (57.1)/6 (42.9)
ABL kinase domain	
*V299L*	1
None	13
Combination of other mutations	
*IKZF1*	3
*HOX11*	2
*ETV6*	2
*TP53*	1
*RUNX1*	2
*ASXL1*	1
*KMT2D*	1
*PHF6*	1
Cytogenetics at diagnosis	
Isolated Philadelphia chromosome	3 (21.4)
Combination of −7	3 (21.4)
Complex karyotype of chromosome	8 (57.2)
Previous therapy of TKIs	
Imatinib	7
Dasatinib	6
Flumatinib	4
At least two kinds of TKI	3
Immediate prior TKIs	
Imatinib	4 (28.6)
Dasatinib	6 (42.8)
Flumatinib	4 (28.6)
Prior CD19 CAR‐T cell/BiTE therapy	2 (14.3)
Prior Ven involved therapy	8 (57.1)
Prior auto‐HSCT	4 (28.6)
Number of prior therapies, median (range)	
Refractory/relapsed group	7 (6–10)
MRD+ group	3 (3–5)
Disease status before olverembatinib+INO	
Hematology relapse	5 (35.7)
MRD persistent positive/relapse	9 (64.3)
Cycles of olverembatinib+ INO	
1	13 (92.9)
2	1 (7.1)
Treatment response	
Complete remission (CR)	14/14 (100)
Complete cytogenetic response	14/14 (100)
Complete molecular response	11/14 (78.6)
MRD negative by flow cytometry	14/14 (100)
Conditioning regimen	
TBI + Cy	6/9
Mel+Cy + Ara‐C + Cla	2/9
Mel+Bu + Cy	1/9

*Note*: Categorical variables are presented as number (percentiles); continuous variables are presented as median (interquartile range) unless otherwise stated.

Abbreviations: Ara‐C, cytarabine; auto‐HSCT, autologous hematopoietic stem cell transplantation; BiTE, bispecific T‐cell engager (blinatumomab); Bu, busulfan; CAR‐T cell, chimeric antigen receptor T cell; Cla, cladribine; Cy, cyclophosphamide; INO, inotuzumab ozogamicin; Mel, melphalan; TBI, total body irradiation; TKI, tyrosine kinase inhibitor; WBC, white blood cell.

**FIGURE 1:**
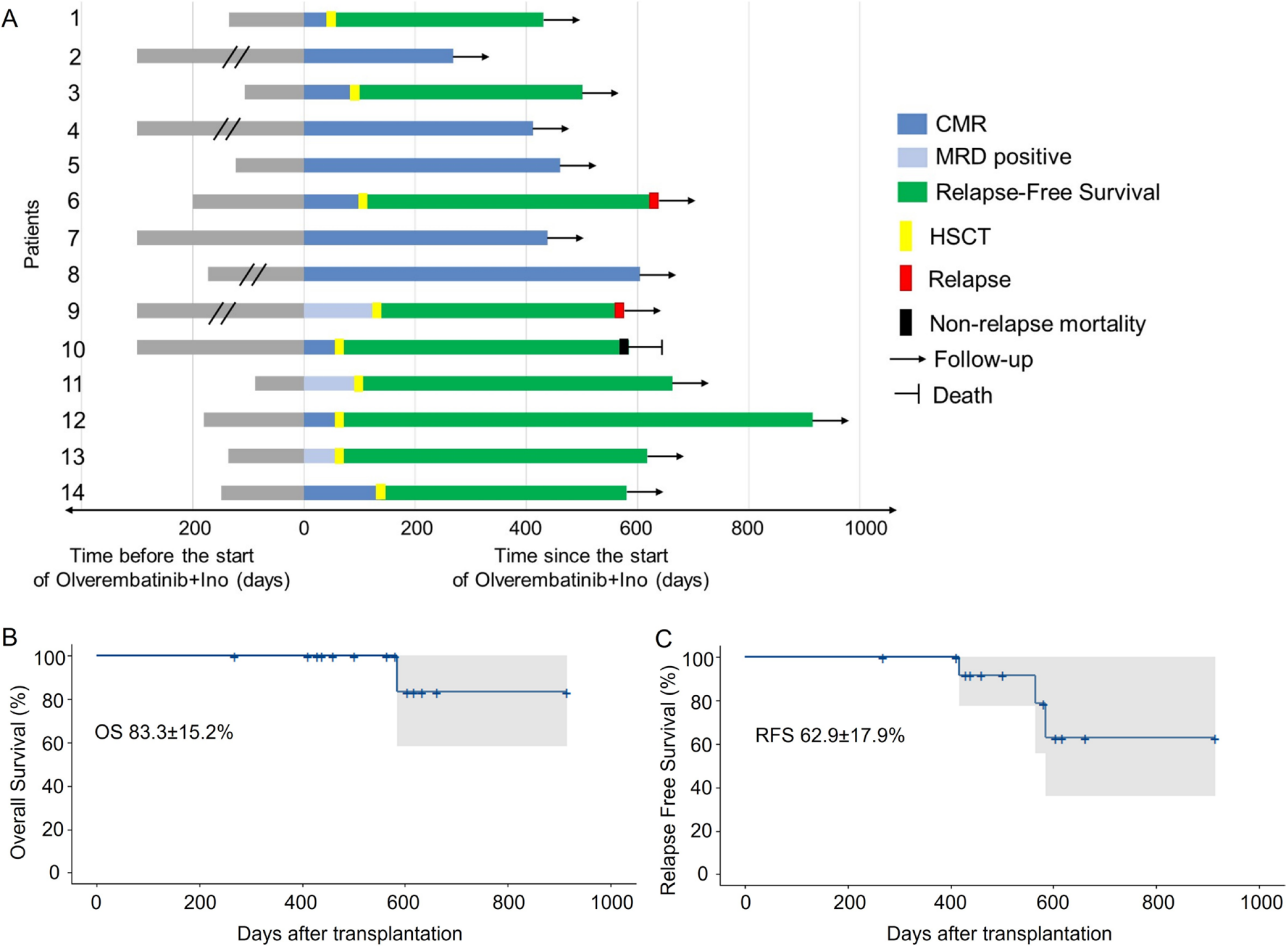
(A). The swimming plot shows the clinical outcomes of the 14 patients in this study receiving olverembatinib and Inotuzumab Ozogamicin treatment. Survival outcomes of patients enrolled, Kaplan–Meier curves for Overall survival (B), Relapse‐free survival (C).

With a median follow‐up time of 564 (268–916) days after treatment, the 2‐year OS rate and recurrence‐free survival rate were 83.3% ± 15.2% and 62.9% ± 17.9%, respectively. Nine patients (64.3%) successfully underwent bridged HSCT (one autologous HSCT (auto‐HSCT) and eight allo‐HSCT) with no cases of veno‐occlusive disease and a 100‐day post‐transplantation mortality of 0%. Six of these patients achieved a CMR prior to allo‐SCT. The median time to allo‐SCT after starting treatment was 93 days (range 52–138). All allogeneic stem cells were from related donors, with 2 HLA‐matched sibling donors and 7 HLA haploidentical donors. The dominant conditioning regimen was total body irradiation (TBI)‐based therapy (*n* = 6, 66.7%). Hematopoietic stem cells were collected from the peripheral blood. Myeloid engraftment and platelet engraftment were successfully achieved for all patients. No primary engraftment failure was observed. Eight patients received maintenance therapy with olverembatinib within 120 days after transplantation, and one patient received maintenance therapy with flumatinib due to economic constraints. For the remaining five patients who did not undergo transplantation, the reasons were failure to effectively control infection and inability to find suitable donors. For patients who did not undergo bridged transplantation, the subsequent treatment options included maintenance therapy with olverembatinib monotherapy (*n* = 1), olverembatinib combined with BiTE/INO (*n* = 2), or olverembatinib combined with maintenance chemotherapy (*n* = 2).

Overall, 2 of the 14 responding patients experienced relapse, including morphological relapse (*n* = 1) and central nervous system leukemia relapse (*n* = 1), at d435 and d461 after allo‐HSCT, respectively. Due to financial limitations, the patient developing hematological relapse declined olverembatinib maintenance treatment post‐HSCT. Additionally, one non‐relapsed patient died of viral encephalitis on d585 after transplantation.

Favorable tolerance and safety of olverembatinib‐INO treatment were demonstrated. Polyserous cavity effusion (*n* = 3), subcutaneous edema (*n* = 1), cardiac arrhythmia (*n* = 2), nausea (*n* = 1), hyperbilirubinemia (*n* = 1), respiratory infection (*n* = 1), and dizziness (*n* = 1) were observed. There were no cases of veno‐occlusive disease with this regimen. All patients completed the treatment, and there were no cases of treatment suspension or discontinuation owing to drug intolerance.

Despite recent progress in TKI and immunotherapies, the prognosis of R/R or persistent MRD Ph + ALL remains dismal. Our findings demonstrate that the combination of olverembatinib and INO represents a safe and potent treatment strategy for this patient population. This combination achieved high rates of both morphological and molecular responses, accompanied by a tolerable toxicity profile.

Olverembatinib, the only third‐generation TKI available in mainland China, exhibits potent activity against the BCR/ABL fusion gene and effectively addresses drug resistance, including the T315I mutation. However, the clinical evidence from published articles for olverembatinib use in Ph + ALL is insufficient. Xiaolan et al. reported an 80% CMR rate in relapsed pediatric Ph + ALL patients [6]. Another study revealed a 71.4% CR rate in R/R ALL patients, 60.0% of whom achieved MRD negativity according to flow cytometry and 47.1% of whom achieved a CMR among the persistently positive MRD patients [7]. Patients were reported to have comparable survival to those treated with ponatinib. In our study, all R/R patients achieved a CR, even a CMR, after only one cycle of treatment. This remarkable outcome is, to a certain extent, attributable to the switch to a third‐generation TKI. On the other hand, the outcome is also dependent on the combined administration of CD22 immunotherapy.

Immunotherapies targeting CD19 or CD22 are essential for R/R B‐cell ALL (B‐ALL). Recently, research has focused on expanding the use of INO to B‐ALL, including in frontline treatment and MRD clearance. In the INO‐VATE study, 81% of the responders had MRD negativity [8]. Moreover, the combination of INO and a TKI has led to an optimization of outcomes. In a phase I/II trial, the combination of INO and bosutinib in the treatment of Ph + ALL resulted in an 83% CR rate [9]. Considering the results of our study and the previously published results, such treatment is very promising. These findings indicate that chemotherapy‐free regimens are promising and that more clinical trials are needed to optimize treatment strategies. However, due to limited numbers and the established promising efficacy of INO monotherapy, larger studies are needed to decipher the benefit of adding Olverematinib to INO.

In summary, our study demonstrated that the use of olverembatinib in combination with INO prior to transplantation is effective and safe for treating R/R‐ or persistent MRD positive Ph + ALL. Treatment with olverembatinib + INO resulted in a high CMR rate, a high bridging transplantation rate, and favorable tolerance. Nevertheless, the findings of this study need to be verified by expanding the number of patients included and conducting prospective clinical studies.

## Author Contributions

X.Z. performed the research, analyzed the data, and wrote the paper. Y.C., J.W., W.Z., Q.M., C.L., X.C., W.C. collected patients' data, managed the database, and contributed essential reagents or tools D.Y., A.P., Y.H., S.F., M.H. critically edited the manuscript. R.Z. and E.J. designed the research study, oversaw the research, and critically reviewed the manuscript. All authors gave final approval for the manuscript.

## Ethics Statement

This study was approved by the Ethics Committee of the Institute of Hematology and Blood Diseases Hospital.

## Consent

All participants signed an informed consent statement prior to participation.

## Conflicts of Interest

The authors declare no conflicts of interest.

## Supporting information


**Data S1:** Supporting Information.

## Data Availability

The data that support the findings of this study are available on request from the corresponding author. The data are not publicly available due to privacy or ethical restrictions.

## References

[1] R. Foà and S. Chiaretti , “Philadelphia Chromosome‐Positive Acute Lymphoblastic Leukemia,” New England Journal of Medicine 386, no. 25 (2022): 2399–2411. 35731654 10.1056/NEJMra2113347

[2] D. A. Berry , S. Zhou , H. Higley , et al., “Association of Minimal Residual Disease With Clinical Outcome in Pediatric and Adult Acute Lymphoblastic Leukemia: A Meta‐Analysis,” JAMA Oncology 3 (2017): e170580. 28494052 10.1001/jamaoncol.2017.0580 PMC5824235

[3] E. Jabbour , N. J. Short , N. Jain , et al., “The Evolution of Acute Lymphoblastic Leukemia Research and Therapy at MD Anderson Over Four Decades,” Journal of Hematology & Oncology 16, no. 1 (2023): 22. 36927623 10.1186/s13045-023-01409-5 PMC10018889

[4] E. Jabbour , F. G. Haddad , N. J. Short , and H. Kantarjian , “Treatment of Adults With Philadelphia Chromosome‐Positive Acute Lymphoblastic Leukemia‐From Intensive Chemotherapy Combinations to Chemotherapy‐Free Regimens: A Review,” JAMA Oncology 8, no. 9 (2022): 1340–1348. 35834222 10.1001/jamaoncol.2022.2398

[5] E. Jabbour , V. G. Oehler , P. B. Koller , et al., “Olverembatinib After Failure of Tyrosine Kinase Inhibitors, Including Ponatinib or Asciminib: A Phase 1b Randomized Clinical Trial,” JAMA Oncology 11, no. 1 (2025): 28–35. 39570620 10.1001/jamaoncol.2024.5157 PMC11583018

[6] X. Li , J. Zhang , F. Liu , et al., “Olverembatinib Treatment in Pediatric Patients With Relapsed Philadelphia‐Chromosome‐Positive Acute Lymphoblastic Leukemia,” Clinical Lymphoma, Myeloma & Leukemia 23, no. 9 (2023): 660–666. 10.1016/j.clml.2023.04.012 37301632

[7] W. Liu , C. Wang , W. Ouyang , et al., “Efficacy and Safety of Olverembatinib in Adult BCR::ABL1‐Positive ALL With T315I Mutation or Relapsed/Refractory Disease,” British Journal of Haematology 205, no. 6 (2024): 2228–2233. 39363594 10.1111/bjh.19804

[8] W. Stock , G. Martinelli , M. Stelljes , et al., “Efficacy of Inotuzumab Ozogamicin in Patients With Philadelphia Chromosome‐Positive Relapsed/Refractory Acute Lymphoblastic Leukemia,” Cancer 127, no. 6 (2021): 905–913. 33231879 10.1002/cncr.33321 PMC7983935

[9] N. Jain , A. Maiti , F. Ravandi , et al., “Inotuzumab Ozogamicin With Bosutinib for Relapsed or Refractory Philadelphia Chromosome Positive Acute Lymphoblastic Leukemia or Lymphoid Blast Phase of Chronic Myeloid Leukemia,” American Journal of Hematology 96, no. 8 (2021): 1000–1007. 33991360 10.1002/ajh.26238 PMC9096877

