# Supplementary material for: Efficacy and Safety of the Third‐Generation Tyrosine Kinase Inhibitor Olverembatinib in Combination With Inotuzumab Ozogamicin for the Treatment of Adult Philadelphia Chromosome‐Positive Acute Lymphoblastic Leukemia Patients With Refractory/Relapsed Disease or Persistent Minimal Residual Disease Bridging to Hematopoietic Stem Cell Transplantation
Source: Am J Hematol. 2025 Aug 6;100(10):1924–8. doi: 10.1002/ajh.70026 (PMC12417767; doi:10.1002/ajh.70026)
Supplement: Supplementary file 1 — Data S1: Supporting Information. [file AJH-100-1924-s001.docx]

**Supplementary materials**

**Method**

**Study design**

Both phase 2 trials were open lable, single arm studies. The first trial (NCT05603156) focused on patients with persistent minimal residual disease, evaluating the efficacy and safety of Inotuzumab Ozogamicin combined with olverembatinib in MRD clearance prior briging to HSCT. The second trial (ChiCTR2200061432) enrolled refractory/relapsed patients, aimed to observe the treatment outcome after allo-HSCT in patients with relapsed and refractory Ph+ALL who was treated with Inotuzumab Ozogamicin combined with olverembatinib achieved CMR.

**Ethics statement**

Both studies were approved by the Ethics Committee of the Institute of Hematology and Blood Diseases Hospital and were conducted in accordance with the guidelines of the Declaration of Helsinki. All patients or their guardians provided written informed consent prior to enrollment.

**Patients**

Study inclusion and exclusion criteria were assessed by the study investigators during patient screening. Study 1 (NCT05603156) enrolled patients with persistent minimal residual disease (MRD) after ≥3 chemotherapy cycles, whereas Study 2 (ChiCTR2200061432) included refractory/relapsed cases. The main inclusion criteria for study participants , apart from requirements of primary disease diagnosis and evaluation, comprised:

aged of at least 16 years, the physical state assessment of Eastern Oncology Collaboration group (ECOG) score ≤3, and adequate organ function assessment, including total bilirubin 1.5 times lower than the upper limit of normal; aspartate aminotransferase (AST) and alanine aminotransferase (ALT) lower than 2 times the upper limit of normal; and serum creatinine <=2 mg/Dl.

The key exclusion criteria included:

1. CD22 was not expressed in lymphocytes with abnormal flow cytometry phenotype;

2. Known HIV or active hepatitis;

3. Suffer from mental illness or other conditions and are unable to cooperate with the requirements of research treatment and monitoring;

4. Pregnant patients or patients unable to take appropriate contraceptive measures during treatment;

5. Suspected allergic to the test drug or any excipients thereof;

6. Active heart disease, defined as one or more of the following:

(1) A history of uncontrolled or symptomatic angina;

(2) Myocardial infarction less than 6 months after enrollment;

(3) A history of arrhythmia requiring drug therapy or severe clinical symptoms;

(4) Uncontrolled or symptomatic congestive heart failure (> NYHA class 2);

(5) The ejection fraction was lower than the lower limit of the normal range;

(6) The patients considered unsuitable for inclusion by the researchers.

**Treatment**

Patients were to recieve a 28 days treatment regimen: Olverembatinib 40mg QOD (d1 to d28 )and Inotuzumab Ozogamicin 1.2mg/m2 (divided into 2 days, d1 and d8, 0.6mg/m2, respectively). MRD was assessed at the end of each treatment cycle. Enrolled patients received a maximum of two treatment cycles before proceeding to HSCT. Post-transplant maintenance treatment with olverembatinib was administered after adequate hematopoietic recovery was achieved under physician supervision. Post-treatment follow-up to assess safety, MRD, relapse, and overall survival continued up to 12 months after treatment.

**End points**

All patients receiving one cycle of treatment were analyzed for efficacy end points. The primary end point was MRD clearance rate. The MRD remission was defined as the fusion gene BCR-ABL quantification in bone marrow samples was lower than the detection limit of RT-PCR (< 0.01%, the residual number of leukemia cells detected in our hospital was 0.0032%) ; at the same time, flow cytometry ( FACS ) was used to monitor the MRD of the cells, and the proportion of abnormal cells < 0.1 % was MRD negative. The key secondary end points evaluated the rate of bridging to HSCT, overall survival, and relapse free survival after HSCT.
